# OLDER ADULT MALNUTRITION: A GROWING CRISIS

**DOI:** 10.1093/geroni/igz038.259

**Published:** 2019-11-08

**Authors:** Meredith Whitmire

**Affiliations:** Matz, Blancato and Associates, Washington, District of Columbia, United States

## Abstract

Malnutrition, a caloric or nutrient imbalance, is particularly common in the older adult population, and food insecurity is a rising concern. This paper will discuss the incidence of malnutrition and food insecurity in the older adult population. It will also discuss federal and state programs in place that work to prevent and combat malnutrition. It will provide strategies for aging network members to engage in these policy discussions.

